# CROSS-SECTOR COLLABORATION TO BUILD DEMENTIA-FRIENDLY BUSINESSES: ENGAGING STUDENTS IN PUBLIC ISSUES IN AGING

**DOI:** 10.1093/geroni/igz038.2660

**Published:** 2019-11-08

**Authors:** Lesa L Huber, Amanda Mosier, Victoria Abramenaka-Lachheb

**Affiliations:** 1 Indiana University School of Public Health, Bloomington, Indiana, United States; 2 IUI Health, IU Health, United States; 3 Indiana University, Bloomington, Indiana, United States

## Abstract

Despite a nationwide campaign to build dementia friendly communities, increasing awareness and engagement with programs like Dementia Friendly Businesses remains a challenge for local champions. To create welcoming environments for people living with dementia (PLWD), communities may benefit from cross-sector collaboration. Fundamental to collaborative efforts is the conviction that individuals, families, businesses, organizations, and institutions each play a role in reducing the stigma associated with dementia and facilitating welcoming environments. This exploratory study considers the outcomes of a cross-sector collaboration between a university, a hospital, individual community members, and students to build awareness of the need for new norms that reduce stigma for people living with dementia. We measured change at each ecological level. Through this collaboration, 61 local businesses and organizations are now DFB certified, 350 individuals have participated in training, and 150 students have joined 12 community trainers to advocate for dementia friendly spaces. Students’ survey responses show that 75% agree or strongly agree that the project engaged them in a meaningful real world experience and advocating for the need for dementia friendly spaces. PLWD provide qualitative data on feeling welcomed in the community. The university and hospital co-created an online open access dementia friendly training taken by 89 people in 6 months. 150 community members came together for a day-long workshop to build capacity and innovate solutions. Implications of the project suggest that changing cultural norms about dementia requires creative solutions that meaningfully engage individuals, organizations, institutions, and communities to collaborate across multiple sectors

